# Metaheuristic-driven dual-layer model for classifying Alzheimer's disease stages

**DOI:** 10.3389/fncom.2026.1731812

**Published:** 2026-02-03

**Authors:** Luka Anicin, Svetlana Andjelic, Marija Markovic Blagojevic, Dejan Bulaja, Miodrag Zivkovic, Tamara Zivkovic, Milos Antonijevic, Nebojsa Bacanin

**Affiliations:** 1 Faculty of Informatics and Computing, Singidunum University, Belgrade, Serbia; 2 Saveetha School of Engineering, Saveetha Institute of Medical and Technical Sciences (SIMATS), Thandalam, Chennai, Tamilnadu, India

**Keywords:** Alzheimer's disease, convolutional neural networks, LightGBM, machine learning, metaheuristics algorithms, MRI, variable neighborhood search, XGBoost

## Abstract

**Introduction:**

Accurate determination of the progression phase of Alzheimer's disease (AD) is crucial for timely clinical decision-making, improved patient management, and personalized therapeutic interventions. However, reliably distinguishing between multiple disease stages using neuroimaging data remains a challenging task.

**Methods:**

This study proposes an advanced machine learning framework for multi-stage AD classification using magnetic resonance imaging (MRI) data. The architecture follows a two-tier design. In the first stage, convolutional neural networks (CNNs) are employed to extract deep and discriminative feature representations from MRI images. In the second stage, these features are classified using ensemble learning models, specifically XGBoost and LightGBM. Metaheuristic optimization strategies are applied to further enhance model performance. The proposed framework was evaluated using a publicly available Alzheimer's disease dataset under three different experimental configurations.

**Results:**

Experimental results demonstrate that the proposed approach effectively addresses the multi-class classification problem across different AD progression stages. The optimized models achieved a maximum classification accuracy of 89.55%, indicating robust predictive performance and strong generalization capability.

**Discussion:**

To improve transparency and clinical relevance, explainable artificial intelligence (XAI) techniques were incorporated to interpret model predictions and highlight feature importance. The results provide meaningful insights into neuroimaging biomarkers associated with AD progression and support the development of more interpretable and trustworthy diagnostic systems. Overall, the proposed framework contributes to improved data-driven decision support and offers a promising direction for future Alzheimer's disease diagnosis and staging research.

## 1 Introduction

Alzheimer's disease (AD) is one of the most debilitating neurodegenerative diseases of modern times, progressively affecting memory, cognition, and the ability to perform routine tasks. Data from the World Health Organization indicate that approximately 57 million people worldwide currently live with dementia, with AD accounting for nearly 60%–70% of all reported cases. Each year, millions of new diagnoses are registered^1^ (Rajan et al., 2021). These statistics emphasize the urgent need for more effective techniques capable of identifying the disease in its earliest phases and tracking its evolution with improved accuracy and consistency (Zhang et al., 2025).

Neuropathological evidence suggests that AD-associated brain degeneration can begin in midlife, although clinical manifestations typically emerge after the age of 65. As the global elderly population continues to expand, the incidence of AD is increasing at an alarming rate (Tajahmadi et al., 2023). Present diagnostic practices combine neurological assessments, cognitive and psychometric evaluations, neuroimaging modalities such as magnetic resonance imaging (MRI) and positron emission tomography (PET), together with cerebrospinal fluid and blood biomarker testing. However, these approaches are often expensive, time-consuming, and inefficient, revealing the pressing demand for more rapid and reliable diagnostic methodologies. The difficulty is particularly evident when trying to recognize the early onset of AD or mild cognitive impairment (MCI), where precise detection remains both challenging and essential to enable timely therapeutic interventions, preventive measures, and support mechanisms that aid patients and caregivers (Prasath and Sumathi, 2023).

Reliable classification of AD progression requires an exact evaluation of cerebral morphology, particularly through volumetric measurements. Although manual segmentation techniques can produce high precision, they are extremely tedious and unfeasible for large-scale implementation, leading to a transition to automated computational strategies for both clinical and research environments (Guenette et al., 2018). Within this framework, the adoption of artificial intelligence (AI) in healthcare has substantially improved diagnostic accuracy, enhanced treatment planning, and facilitated more effective healthcare delivery by reducing expenses and improving patient outcomes (Raza et al., 2025). As a result, AD categorization has become a vibrant area of scientific inquiry over the past decade. A considerable number of contemporary studies focus on deep learning (DL), most notably convolutional neural networks (CNNs) (Gu et al., 2018), which have become the dominant architecture, while others employ more classical machine learning (ML) techniques. CNNs have exhibited remarkable capability to capture distinctive patterns from neuroimaging data such as magnetic resonance imaging and PET. Similarly, gradient boosting algorithms including AdaBoost (Hastie et al., 2009) and CatBoost (Prokhorenkova et al., 2018) have achieved competitive results when working with structured datasets. Numerous investigations have reported impressive success using CNNs for AD stage prediction (Shamrat et al., 2023; Degadwala et al., 2023), while others have explored alternative ML paradigms (Nawaz et al., 2021).

Despite their broad application, ML-driven models face several fundamental obstacles. Their performance can severely degrade due to issues like biased or low-quality data, suboptimal algorithm selection, and inadequate hyperparameter adjustment. Models trained on imbalanced, noisy, or poorly curated datasets often produce erratic and unreliable output, underscoring the importance of developing high-quality and representative training collections. Furthermore, the efficiency of each ML algorithm is inherently context-dependent, and individual models may demonstrate drastically different effectiveness depending on the specific dataset and problem domain. Hyperparameters also exert a critical role, since determining their ideal configurations demands systematic and often computationally demanding fine-tuning. This difficulty aligns with Wolpert's no free lunch (NFL) theorem (Wolpert and Macready, 1997), which asserts that no single algorithm outperforms all others in all types of problem. Consequently, each method must be adapted and optimized for its different applications. Nevertheless, the hyperparameter optimization process itself is notoriously intricate and is generally regarded as NP-hard. As both data complexity and the dimensionality of the search space increase, identifying near-optimal configurations becomes computationally burdensome and frequently infeasible. Traditional optimization approaches often fail to yield satisfactory results under such challenging circumstances.

To overcome these limitations, metaheuristic optimization techniques have emerged as a potent alternative. These algorithms are particularly proficient in traversing vast and complex search landscapes to approximate optimal solutions when exact optimization becomes computationally unattainable. Due to their flexibility and effectiveness, metaheuristics are especially advantageous for hyperparameter tuning. By producing high-quality approximations, they substantially improve the performance and robustness of ML models across a broad range of practical domains.

To address the challenge of categorization of the AD stage, this research proposes a novel dual-layered framework inspired by methodologies that have demonstrated outstanding results in domains such as software testing (Petrovic et al., 2024; Villoth J. P. et al., 2025), intrusion detection (Antonijevic et al., 2025), and web security improvement (Jovanovic et al., 2023). In the first stage, a CNN is used to extract distinctive and meaningful features from the MRI scans. Building upon earlier studies (Petrovic et al., 2024; Villoth J. P. et al., 2025) that showed how replacing the final dense layer of CNNs with advanced ensemble learners can considerably improve model accuracy, the proposed framework substitutes this concluding layer with XGBoost (Chen and Guestrin, 2016) and LightGBM (Ke et al., 2017) classifiers.

Rather than relying exclusively on CNN-based end-to-end classification, the proposed architecture leverages the convolutional layers for hierarchical feature abstraction, after which the obtained deep representations are passed to a secondary classification phase handled by ensemble algorithms. To further enhance the overall performance of the model, metaheuristic optimization techniques are integrated to fine-tune hyperparameters at both levels, ensuring optimal adjustment of the CNN feature extractor and the ensemble classifiers. This hybrid architecture combines CNN ability to capture complex features, the robust decision fusion capacity of gradient boosting models, and the adaptive exploration efficiency of metaheuristics. The synergy achieved through this integration of deep learning, ensemble-based classification, and intelligent hyperparameter optimization results in improved predictive performance and increased computational efficiency in AD stage detection.

In the proposed model, hyperparameter tuning is carried out through a custom variation of the well-known variable neighborhood search (VNS) algorithm (Mladenović and Hansen, 1997). The selection of VNS followed extensive comparative experiments involving multiple optimization techniques, consistent with the rationale of the NFL theorem (Wolpert and Macready, 1997), which states that no single optimizer consistently outperforms all others in every class of problems. Although several other state-of-the-art metaheuristic approaches were also tested, preliminary experiments on smaller AD classification datasets indicated that VNS consistently achieved stable and high-quality solutions. These findings highlighted the robustness, adaptability, and suitability of the algorithm for complex optimization landscapes, motivating its implementation as the primary optimization mechanism in this study. By adapting VNS to the specific needs of AD stage prediction, the framework achieves more efficient tuning of both CNN and ensemble components, leading to superior predictive accuracy and higher reliability of the system.

Moreover, this research fills an important methodological gap, as the integration of CNN-based feature extraction with gradient boosting classifiers within a coordinated, multi-tiered framework refined through advanced metaheuristic optimization has not yet been systematically explored for this particular task. Taking into consideration all these aspects, the main methodological innovations and novel contributions of this work can be summarized as follows:

- Development of a hybrid AI-based analytical framework that combines feature extraction, deep learning, and conventional machine learning methods, specifically designed for accurate classification of Alzheimer's disease stages based on MRI-derived data.
- Construction of a two-phase classification strategy in which CNNs are used to hierarchically extract deep neuroimaging features, which are subsequently refined through classical ML algorithms to achieve precise differentiation among AD stages.
- Implementation of computationally efficient models that utilize lightweight CNN architectures coupled with shallow XGBoost and LightGBM classifiers, each optimized with minimal hyperparameter complexity, thus allowing potential deployment in low-resource settings such as embedded systems and portable diagnostic platforms.
- Formulation of a customized optimization method inspired by the standard VNS algorithm, specifically adapted to systematically fine-tune the network and classifier parameters, thus improving the classification precision at both hierarchical levels of the proposed system.
- Incorporation of explainable artificial intelligence (XAI) techniques to ensure transparent interpretation of the model's decision-making process, focusing on importance of characteristics and contribution analysis.

The remainder of this paper is organized as follows. Section 2 introduces the fundamental theoretical background and reviews the principal methodological paradigms that serve as the basis for the proposed framework. Section 3 provides a detailed description of the algorithmic design and explains the two-stage classification approach developed to identify the progression of AD using MRI data. Section 4 outlines the complete experimental configuration, including all parameter settings necessary to guaranty full reproducibility. Section 5 reports the empirical results obtained from the experiments conducted, while Section 6 presents a comprehensive statistical assessment and interpretive discussion of these results. Finally, Section 7 summarizes the main contributions of this study and suggests possible directions for future research within the field.

## 2 Related works

AD is a progressive neurodegenerative condition characterized by a steady deterioration in memory, cognitive performance, and behavioral control. Early and accurate detection of AD is widely regarded as fundamental for the successful clinical management and design of targeted therapeutic interventions (Singh et al., 2024). During the past decade, ML and DL techniques have emerged as transformative approaches to identify and stage AD, utilizing multimodal sources such as magnetic resonance imaging, PET, and biochemical biomarkers. Recent computational and electrophysiological studies have contributed substantive insights into AD mechanisms relevant to this problem. For example, Kaushik et al. (2024) developed a computational model of hippocampal pyramidal neurons to investigate how β-amyloid-induced disruptions in calcium-dependent ionic channels affect theta rhythm dynamics, linking ionic dysregulation to functional impairment in memory-related neural circuits. Complementing such modeling approaches, studies like (Babiloni et al., 2020; Yu et al., 2021; Costanzo et al., 2024) reviewed the role of electrophysiological biomarkers, including EEG and MEG, in characterizing neural synchronization and connectivity changes associated with Alzheimer's pathology, underscoring the value of real-time neurophysiological measurements for understanding disease progression and potential diagnostic markers.

A growing body of literature underscores the value of hybrid analytical frameworks and feature-driven deep models, which have significantly improved diagnostic accuracy (Chen et al., 2022). For example, Arya et al. (2023) conducted an extensive review of ML and DL-based approaches to differentiate cognitively normal individuals from AD patients in the early stages of the disease. Their findings identified MRI and PET as the most commonly applied imaging modalities and compared classification performance between various algorithms. Similarly, Zhao et al. (2023) analyzed the comparative effectiveness of traditional ML methods for the prediction of AD using MRI data. Their evaluation included support vector machines, random forests, CNNs, autoencoders, and transformer-based models, addressing trade-offs between preprocessing pipelines, conventional ML methods, and modern DL architectures. In addition, they discussed the advantages and limitations of different input representations, offering valuable insights into the development of more effective AD diagnostic models.

In another example, Helaly et al. (2022) introduced a framework aimed at the early detection and stage-specific categorization of AD from medical images. Their method employed CNNs to perform pairwise binary classifications between AD stages, effectively decomposing the multi-class classification problem into smaller binary tasks. Two methodological configurations were analyzed: one used standard CNN models to process both 2D and 3D neuroimaging data, while the other leveraged transfer learning with pre-trained networks such as VGG19 to enhance prediction accuracy. In a complementary direction, Sarkar (2025) explored the integration of deep learning with gait analysis to improve diagnostic robustness. They combined CNNs and recurrent neural networks (RNNs) to differentiate between cognitively healthy individuals and those at risk using motion data collected from wearable sensors and motion capture technologies, highlighting the potential of non-invasive, movement-based biomarkers in early AD detection.

A continuing issue in DL-based AD diagnostics is their tendency to operate as opaque black box systems, producing outputs without clear interpretability. To confront this limitation, Bloch et al. (2024) conducted a systematic investigation to improve the transparency of the model by identifying the neuroanatomical regions activated during inference and comparing these with the interpretability output of traditional ML models. Their work used a wide range of explainability techniques, providing a thorough assessment of interpretability within AD diagnostic systems. Similarly, Menagadevi et al. (2024) stressed the critical role of preprocessing and image enhancement in increasing classification accuracy. Their review discussed key MRI preprocessing steps such as denoising, illumination normalization, and intensity correction, followed by segmentation techniques to isolate regions of interest, feature extraction methods, and the application of various ML and DL algorithms for AD classification, thus presenting a comprehensive methodological overview from data preparation to classification.

Beyond the binary challenge of distinguishing the presence of AD, stratification of disease progression stages has become a prominent research focus. Both deep learning and conventional ML techniques generally require large datasets to form stable feature representations; however, this necessity introduces issues such as overfitting and class imbalance. To mitigate these challenges, several studies have adopted transfer learning and hybrid modeling strategies. For example, Nawaz et al. (2021) developed a deep feature-based AD staging approach, where features extracted from a pre-trained AlexNet model were subsequently classified using traditional ML algorithms like random forests, k-nearest neighbors and support vector machines. Similarly, Nguyen et al. (2022) proposed an ensemble model that merged deep and traditional learning, employing a 3D-ResNet to capture volumetric MRI patterns and an XGBoost classifier to identify discriminative voxel-level signals. Another approach was presented in Mahanty et al. (2024b), where the authors developed an ensemble DL approach using an enhanced Xception model and snapshot blending to achieve highly accurate multi-class AD detection from brain MRI scans. Transfer-learning models were examined in Mahanty et al. (2024a) to classify AD from medical imaging data, demonstrating improved detection performance compared to individual models.

Further advancing this direction, El-Sappagh et al. (2022) introduced a two-phase multimodal DL framework to track AD progression. The first stage used multiclass classification to assign diagnostic labels, while the second applied regression analysis to estimate the time-to-conversion from mild cognitive impairment (MCI) to AD, providing both categorical and temporal insight. Building on that work, El-Assy et al. (2024) presented a CNN-based system trained on MRI scans that utilized two separate CNN branches with distinct kernel dimensions and pooling strategies, integrated through a shared output layer to facilitate multi-class categorization across three to five disease stages.

Additional research has focused on refining CNN architectures for more granular disease stratification. Savaş (2022) evaluated 29 pre-trained CNN networks to classify MRI scans into three categories: cognitively normal, moderate cognitive impairment, and AD. Extending these findings, Shamrat et al. (2023) developed AlzheimerNet, a specialized CNN architecture capable of differentiating between five stages of AD in addition to a control group. Their approach incorporated contrast limited adaptive histogram equalization (CLAHE) to improve MRI image quality prior to classification. Finally, Givian et al. (2024) proposed a feature-based ML framework that employs structural MRI features with several classifiers, including random forests, k-nearest neighbors, support vector machines, decision trees, and multilayer perceptrons, to segment disease phases, thus offering comparative insights into the respective strengths of traditional ML and deep feature-based methods.

### 2.1 Technology background

CNNs (Gu et al., 2018) have become one of the most transformative architectures in artificial intelligence, largely due to their outstanding capabilities in image classification, pattern recognition, and object detection. Over time, their use has expanded far beyond visual perception, extending into fields such as natural language processing, biomedical imaging, and environmental modeling. The conceptual basis of CNNs draws inspiration from the hierarchical organization of the mammalian visual cortex, in which sensory information is processed through successive layers of increasing abstraction. In artificial models, this hierarchical mechanism is reproduced as the data move through multiple interconnected layers, where nonlinear activation functions, such as the rectified linear unit (ReLU), hyperbolic tangent (tanh), and sigmoid, allow the network to model complex nonlinear relationships among features.

A typical deep CNN is composed of several distinct types of layers: convolutional, activation, pooling, and fully connected layers. In the convolutional layer, a set of trainable filters (kernels) systematically traverse the input, performing localized dot-product computations between filter weights and corresponding input regions. The result is a group of feature maps that capture local patterns and spatial hierarchies. These feature maps are subsequently passed through activation layers, which introduce the nonlinearity necessary for learning complex dependencies. Among all activation functions, ReLU is the most widely used due to its computational simplicity and effectiveness in mitigating the vanishing gradient issue (Nair and Hinton, 2010).

The pooling layers perform spatial subsampling to reduce the dimensionality of the feature map while preserving the most important information. Max pooling, the most common approach, selects the highest value within each neighborhood, usually achieving a reduction of 70% to 80% in dimensionality without a considerable loss of relevant information. The abstract, high-level features obtained after a series of convolutional and pooling stages are finally processed by fully connected layers, which act as the decision-making component of the network, transforming learned features into the final class predictions.

CNNs have shown exceptional flexibility in a wide range of computer vision tasks (Bhatt et al., 2021), including face recognition (Budiman et al., 2023), document and handwriting analysis (Hasib et al., 2023), and medical image classification for diagnosis of diseases and clinical screening (Salehi et al., 2023; Purkovic et al., 2024). Beyond medical applications, CNN architectures have also been used successfully in climate and environmental studies, particularly to model global weather dynamics and predict extreme meteorological events (Kareem et al., 2021).

XGBoost (Extreme Gradient Boosting) is a machine learning algorithm based on high-performance gradient boosting (Chen and Guestrin, 2016). It constructs a ensemble of decision trees in a sequential manner, where each new tree corrects the errors of the preceding, resulting in enhanced predictive precision and model robustness. Recognized for its scalability and speed, XGBoost incorporates regularization to reduce overfitting and supports parallelized learning, making it well-suited for large, high-dimensional datasets. Its adaptability allows it to handle both classification and regression tasks, with several tunable hyperparameters that significantly affect performance. Thanks to its efficiency, reliability, and interpretability, XGBoost is widely adopted in cybersecurity, IoT data analytics, and other real-world applications that require fast and accurate data-driven predictions.

LightGBM (Ke et al., 2017), an open-source framework developed by Microsoft, is specifically designed for large-scale high-speed data processing. Its efficiency arises from techniques such as Gradient-based One-Side Sampling (GOSS), which preserves samples with larger gradient magnitudes to maintain accuracy, and Exclusive Feature Bundling (EFB), which combines mutually exclusive features to reduce dimensionality and computational load. These mechanisms allow LightGBM to train significantly faster and with lower memory consumption than traditional boosting algorithms, making it highly effective for massive datasets with numerous features.

This framework has proven reliable in a range of predictive problems, including classification, regression, and anomaly detection, and has found applications in structural analysis (Li et al., 2023), financial prediction (Wang et al., 2022), and defect identification (Lao et al., 2023). LightGBM also supports parallel and distributed computation, enabling seamless scalability in modern computing environments. Its main hyperparameters, such as the number of leaves per tree, the maximum depth of the tree, and the learning rate, play an essential role in determining overall model performance and predictive capacity.

### 2.2 Metaheuristics optimization

A persistent and fundamental challenge in machine learning lies in the optimization of hyperparameters, a task widely acknowledged as NP-hard because of its immense combinatorial search space and computational complexity. This difficulty is further reinforced by the NFL theorem (Wolpert and Macready, 1997), which states that no single optimization approach can consistently outperform all others in all category of problems, as its effectiveness is inherently tied to the characteristics of the data set, the performance metrics, and the parameter configurations involved.

To mitigate these constraints, increasing attention has been focused toward metaheuristic optimization techniques. Metaheuristics, particularly those inspired by swarm intelligence, constitute a class of stochastic optimization strategies modeled after the collective behaviors observed in natural systems such as flocks of birds, swarms of insects, and herds of animals. These methods are particularly well-suited for solving complex, NP-hard problems because they maintain a dynamic balance between global exploration of the search space and local exploitation of promising regions. Nevertheless, population-based methods often face the drawback of overemphasizing one of these components, which can lead to premature convergence or suboptimal stagnation. To counteract this, hybrid approaches and adaptive mechanisms are frequently employed to preserve equilibrium and enhance the robustness of the search process.

Well-known members of this algorithmic family include particle swarm optimization (PSO) (Kennedy and Eberhart, 1995), genetic algorithm (GA) (Mirjalili, 2019), and numerous nature-inspired variants such as the reptile search algorithm (RSA) (Abualigah et al., 2022), whale optimization algorithm (WOA) (Mirjalili and Lewis, 2016), red fox algorithm (RFA) (Połap and Woźniak, 2021), sine cosine algorithm (SCA) (Mirjalili, 2016), artificial bee colony (ABC) (Karaboga and Basturk, 2007), firefly algorithm (FA) (Yang and He, 2013b), elk herd optimization (EHO) (Al-Betar et al., 2024), variable neighborhood search (VNS) (Mladenović and Hansen, 1997), and COLSHADE (Gurrola-Ramos et al., 2020). Together, these methods form a comprehensive and versatile set of tools capable of addressing diverse and computationally intensive optimization problems across scientific and engineering disciplines.

Metaheuristic approaches have shown strong performance in a variety of domains, including software engineering (Villoth J. P. et al., 2025; Villoth S. J. et al., 2025), medical diagnostics (Zivkovic et al., 2023, 2024), and a range of applied optimization scenarios (Bacanin et al., 2024; Lakicevic et al., 2024; Antonijevic et al., 2024; Petrovic et al., 2025; Bozovic et al., 2025). However, their use in the healthcare sector, particularly in the modeling of neurodegenerative disorders and the classification of stages of AD using neuroimaging, remains relatively underexplored (Antonijevic et al., 2024; Dobrojevic et al., 2024).

Drawing on their proven success in related areas, the integration of metaheuristic algorithms into neurodegenerative disease prediction represents a promising pathway toward enhancing diagnostic precision, model generalization, and individualized clinical evaluation. In this study, a cooperative dual-layer classification framework is proposed, in which a CNN performs hierarchical MRI feature extraction, followed by XGBoost and LightGBM classifiers for refined stage identification. Crucially, metaheuristic optimization is used to tune the hyperparameters in both phases, forming a unified and adaptive strategy that advances an automated and interpretable classification of AD progression.

## 3 Methods

This section begins with an overview of the conventional VNS algorithm. Then it discusses the primary limitations of the original formulation, followed by a detailed explanation of the modified variant developed in this research, and a brief outline of the complete classification framework.

### 3.1 Basic variable neighborhood search algorithm

Local search algorithms in combinatorial optimization improve an initial candidate solution by iteratively exploring its surrounding configurations and replacing it with a better alternative until no further enhancement of the objective function can be obtained. During each iteration, an improved solution *x* is selected from its neighborhood set *N*(*x*), and the search is completed once a local optimal point is reached. Unlike conventional local search methods that follow a single continuous search trajectory, VNS (Mladenović and Hansen, 1997) uses a structured diversification principle. Instead of restricting exploration to a single neighborhood, VNS systematically expands the search to progressively more distant neighborhoods, accepting a new solution only when it provides a measurable improvement. This strategy allows the algorithm to retain the beneficial properties of a near-optimal solution while simultaneously investigating unexplored regions of the search space that may yield superior outcomes. Each newly generated candidate solution is subsequently refined through a local search procedure to promote convergence toward a local optimum.

More precisely, VNS operates using a finite collection of neighborhood structures *N*_*k*_, where *k* = 1, 2, …, *k*_*max*_. The algorithm transitions between these neighborhoods through three main stages:

- A random candidate *x*′ is generated within the current neighborhood *N*_*k*_, helping to reduce the likelihood of premature convergence and redundant search cycles.
- Then a local search is applied to *x*′, producing an improved solution *x*″ that is locally optimal with respect to *N*_*k*_.
- If *x*″ demonstrates improvement compared to the current best solution, it replaces it, and the exploration continues within the same neighborhood; otherwise, the procedure advances to the next neighborhood structure.

The algorithm terminates when the stopping conditions are met, such as when a predefined number of iterations is reached or the computational budget is exhausted.

### 3.2 Modified VNS

Original VNS has been widely recognized as a powerful and adaptable modern generation optimization method that exhibits strong performance across a wide spectrum of application areas. However, despite its reliability and versatility, extensive empirical studies utilizing contemporary benchmark suites (Luo et al., 2022) have identified several limitations, particularly its relatively restricted exploratory capability during the early phases of the optimization process. In addition, the algorithm may occasionally suffer from premature convergence toward local optima, which can negatively impact its overall convergence efficiency under certain conditions.

To overcome these limitations, the first enhancement introduced in this work focuses on increasing population diversity during the initial optimization phase. This improvement is achieved through the integration of the Quasi-Reflexive Learning (QRL) mechanism (Rahnamayan et al., 2007) into the population initialization procedure. In this extended scheme, the initial population is divided into two complementary subsets: one generated using the standard VNS initialization process, and the other constructed through QRL-based diversification. The latter subset expands the spatial coverage of the search space from the outset, reducing the possibility of early clustering among agents and promoting a more uniform and comprehensive exploration of the solution landscape. The mathematical formulation of this quasi-reflexive generation procedure is given in Equation 1, which defines how mirrored solution vectors are produced to supplement their original counterparts.


$$\begin{array}{l} X_{j}^{qr}=rnd(\frac{lb_{j}+ub_{j}}{2},x_{j}) \end{array}$$ (1)

In this formulation, $\frac{lb_{j}+ub_{j}}{2}$ denotes the midpoint between the lower and upper boundaries of the *j*-th dimension in the search space, while *rnd*() produces a random value within the specified interval. QRL therefore generates complementary candidate solutions by probabilistically sampling between the midpoint of the search interval and the current solution, consequently enhancing population diversity during early exploration. A detailed mathematical analysis of this mechanism is provided in the original formulation (Rahnamayan et al., 2007).

The second improvement incorporated into the VNS algorithm introduces a soft rollback mechanism, designed in this research to alleviate convergence stagnation. This mechanism is triggered when the algorithm does not exhibit notable improvement over a defined interval of *T*/3 iterations, where *T* represents the total number of permitted iterations. The value of this threshold was determined empirically. When stagnation occurs, the population is partially reverted to its most recent productive configuration, allowing the algorithm to recover from unproductive search directions. To implement this mechanism, two auxiliary control parameters are introduced: the stagnation counter (*s*_*count*) and the stagnation threshold (*s*_*tresh*), initialized as *s*_*count* = 0 and *s*_*tresh* = *T*/3. The counter increases with every iteration that lacks an improvement in fitness, and when *s*_*count* reaches *s*_*tresh*, the rollback process begins.

This rollback strategy integrates an elitist preservation principle to safeguard the overall quality of solutions. Specifically, the best-performing individual, defined as the candidate who reaches the optimal fitness value, is retained, while the remaining members of the population are regenerated according to the original initialization procedure of the algorithm. This approach effectively restores population diversity without sacrificing the most promising solution identified so far.

To reflect these algorithmic refinements, the proposed variant is named the quasi-reflexive learning stagnation-aware VNS (QSAVNS). The complete step-by-step procedure of this modified method is presented in Algorithm 1.

Algorithm 1 **QSAVNS procedural logic.**

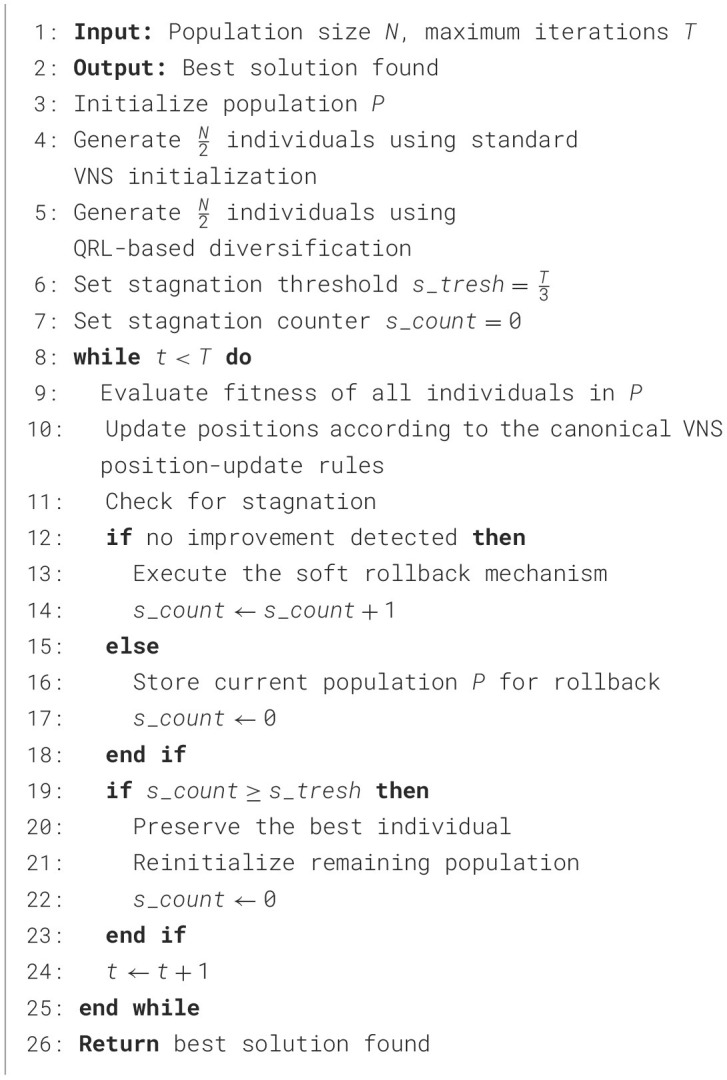

Because elapsed runtime is heavily relying on hardware and implementation specifics, algorithmic complexity of metaheuristics is typically assessed in terms of fitness function evaluations, which is the standard and more reliable measure in metaheuristic optimization research. In each run, the fitness function evaluation (FFE), corresponding to model training and validation for one hyperparameter configuration, is the most computationally expensive operation and therefore dominates the overall runtime of the algorithm. From a computational standpoint, QSAVNS preserves the same fitness-evaluation complexity as baseline VNS. The QRL-based initialization and stagnation-aware rollback only alter solution generation/diversification, while maintaining *N* fitness evaluations within each of *T* iterations. Consequently, the overall complexity remains *O*(*N*×*T*) in FFEs, which is identical to baseline VNS.

### 3.3 Proposed framework

The proposed method operates as the core optimization engine within a two-layer classification architecture. In this design, the hyperparameters of the classifiers' hyperparameters are represented as agent-specific variables, and optimization is carried out iteratively through repeated cycles of model training, parameter adjustment, and performance evaluation until a predefined convergence criterion is satisfied.

At the first level (L1), this iterative optimization is applied to CNNs. Once the most suitable CNN configuration is identified, its final output layer is removed, and the intermediate feature embeddings learned during training are extracted. These representations are subsequently passed to the second level (L2), where ensemble boosting classifiers are employed. In this second stage, the boosting models also undergo metaheuristic optimization, with their hyperparameters encoded as evolutionary traits of the agents within the population.

## 4 Experimental setup

### 4.1 Dataset overview

For this research, a dataset was used from the Kaggle platform.^2^ The dataset was reduced to 10% of its original volume while preserving proportional representation between all classes to maintain balance. It is intended for the classification of AD stages and contains four distinct categories: No Dementia (class 0), Very Mild Dementia (class 1), Mild Dementia (class 2), and Moderate Dementia (class 3). The original dataset was already partitioned into training and testing subsets by class and was utilized in this study in its existing form, without any additional preprocessing or modification.

### 4.2 Evaluation metrics

During the simulation phase, the performance of the model was assessed using a standard set of classification metrics, namely precision, precision, recall, and the F1-score, formally defined in Equations 2–5.


$$\begin{array}{l} Accuracy=\frac{TP+TN}{TP+FP+TN+FN} \end{array}$$ (2)

$$\begin{array}{l} Precision=\frac{TP}{TP+FP} \end{array}$$ (3)

$$\begin{array}{l} Recall=\frac{TP}{TP+FN} \end{array}$$ (4)

$$\begin{array}{l} F1\text{\_}score=\frac{2\cdot Precision\cdot Recall}{Precision+Recall} \end{array}$$ (5)

In the above equations, *TP*, *TN*, *FP*, and *FN* denote the numbers of true positives, true negatives, false positives, and false negatives, respectively.

In addition to conventional classification metrics, the Matthews correlation coefficient (MCC) (Matthews, 1975) was used as an additional evaluation measure. Due to its robustness in handling imbalanced class distributions, the MCC offers a more comprehensive and reliable indication of the overall performance of the model. Its calculation is formally defined in Equation 6.


$$\begin{array}{l} \text{MCC}=\frac{\left(TP\times TN\right)-\left(FP\times FN\right)}{\sqrt{\left(TP+FP\right)\left(TP+FN\right)\left(TN+FP\right)\left(TN+FN\right)}} \end{array}$$ (6)

Across all simulation experiments, the MCC was designated as the main optimization objective with the aim of maximizing its value to obtain the most balanced and accurate classification results.

### 4.3 Experimental setup

A series of three experimental studies was conducted in which metaheuristic optimization algorithms were employed to fine-tune the parameters across both layers of the proposed dual-stage classification framework. The architecture consisted of a CNN as the first-layer module, followed by XGBoost and LightGBM classifiers that form the second-layer classification component. In each experimental scenario, the proposed QSAVNS metaheuristic served as the principal optimization algorithm, and its performance was systematically compared with several well-established optimization methods. The comparison group included the canonical VNS (Mladenović and Hansen, 1997), GA (Mirjalili, 2019), PSO (Kennedy and Eberhart, 1995), ABC (Karaboga and Basturk, 2007), BA (Yang and He, 2013a), SCHO (Bai et al., 2023), and EHO (Al-Betar et al., 2024), providing a representative balance between the classical and more recent optimization paradigms. All competing algorithms were executed using their standard parameter configurations as specified in the original studies. To maintain methodological consistency, identical experimental conditions were applied to each algorithm in all three experiments. To minimize the effect of stochastic variability inherent in metaheuristic processes, each method was independently executed 30 times.

Given the high computational cost of CNN training, the first-layer (L1) experiments used a reduced population of eight candidate solutions (*N* = 8) and a maximum of five iterations per run (*max*_*iter* = 5). For the optimization of XGBoost and LightGBM, the population size was set to ten (*N* = 10), with ten iterations per execution (*max*_*iter* = 10). Within the metaheuristic optimization procedure, each individual in the population encodes a unique configuration of a neural network or ensemble model (CNN, XGBoost, or LightGBM) along with its associated hyperparameters. Evaluating each configuration requires multiple training-validation cycles, which are computationally demanding. To alleviate this burden, the size of the population and the iteration count were deliberately constrained, thus reducing the total number of retraining operations. Furthermore, once the population size exceeds a certain threshold, additional expansion typically produces negligible improvements in optimization performance. Empirical evidence suggests that metaheuristic algorithms can often converge to near-optimal solutions even with moderate population sizes, providing an efficient and resource-conscious approach to solving high-cost optimization tasks. As previously stated, the optimization objective was defined as the maximization of the MCC.

In the first experimental setup, metaheuristic algorithms were applied to optimize the CNN component in the initial layer (L1) of the proposed framework. The tunable CNN hyperparameters, listed in Table 1, were intentionally limited to lightweight configurations to allow potential deployment on resource-constrained platforms such as ESP32 or Arduino. A batch size of 512 was used and an early stopping was triggered after one-third of the total training epochs. The target image resolution was fixed at (32, 32) using the RGB color mode and categorical label encoding. Within the cooperative dual-layer design, the optimized CNN from this stage provided the feature extraction foundation for the subsequent ensemble-based classification phase.

**Table 1:** Model configurations with corresponding optimized hyperparameters and their respective search ranges.

**Model**	**Hyperparameter**	**Low limit**	**High limit**
L1 CNN	Learning rate	0.0001	0.003
	Dropout	0.05	0.2
	Epochs	10	30
	Convolutional layers	1	2
	Dense layers	1	2
	Cells per layer	32	96
L2 XGBoost	Learning rate	0.1	0.9
	Minimum child weight	1	5
	Subsample	0.01	1
	Col sample by tree	0.01	1
	Max depth	1	5
	Gamma	0	0.8
L2 LightGBM	Number of rounds	5	20
	Max depth	3	10
	Number of leaves	3	10
	Minimum child weight	1	5
	Feature fraction	0.1	0.9
	Bagging fraction	0.5	1
	Min split gain	0.001	0.1
	Lambda l1	0	5
	Lambda l2	0	3
	Learning rate	0.01	0.9

In the second experimental configuration, the XGBoost algorithm was applied within the classification layer (L2) of the framework. For this purpose, the intermediate feature embeddings generated by the optimized CNN from the first layer were extracted from the output of the dropout layer and saved for all data samples. Then these characteristic vectors were split into training and testing subsets following a 70%–30% ratio. The resulting feature representation was used as input for both the training and hyperparameter optimization of the XGBoost classifier. The specific parameters selected for tuning, along with their search intervals, are summarized in Table 1.

In the third experimental study, the LightGBM algorithm was integrated into the second layer (L2) of the proposed architecture. The classifier was trained and optimized using the same intermediate feature representations produced by the CNN in the preceding experiment. The LightGBM hyperparameters chosen for optimization, along with their defined search ranges, are also presented in Table 1.

## 5 Simulation results

The experimental analyses concentrated on the integration of CNNs within the first layer (L1) of the proposed framework, where they handled the initial processing and extraction of discriminative features from MRI images corresponding to different stages of AD. In the subsequent layer (L2), gradient boosting classifiers were used to perform the final stage classification. At this level, two competitive boosting models, XGBoost and LightGBM, were utilized, both exhibiting strong and consistently stable performance throughout the evaluation process.

### 5.1 L1 CNN

Table 2 presents a comparative analysis of CNN models optimized using several metaheuristic algorithms, with the MCC serving as the primary objective metric. Among the evaluated methods, the proposed QSAVNS optimizer achieved the best overall result, achieving a maximum MCC of 0.287398. In comparison, the strongest worst-case performance was obtained by SCHO (0.239755), which also produced the highest mean MCC (0.251198) and the best median value (0.249601). Furthermore, it is worth noting that within this experimental configuration, the ABC algorithm exhibited the most consistent behavior, as evidenced by its minimal variance across multiple independent runs.

**Table 2:** Results of the objective and indicator functions obtained during L1 CNN optimization.

**Method**	**Best**	**Worst**	**Mean**	**Median**	**Std**	**Var**
CNN-QSAVNS	0.287398	0	0.156415	0.169131	0.129712	0.016825
CNN-VNS	0.229776	0.080976	0.154687	0.153998	0.060673	0.003681
CNN-GA	0.026130	0	0.006532	0	0.011314	0.000128
CNN-PSO	0.255399	0.212239	0.234623	0.235426	0.019539	0.000382
CNN-ABC	0.244962	0.228073	0.237688	0.238860	0.007073	5.00E-05
CNN-BA	0.248187	0.158546	0.208912	0.214457	0.032671	0.001067
CNN-SCHO	0.265833	0.239755	0.251198	0.249601	0.011586	0.000134
CNN-EHO	0.054054	0	0.018991	0.010954	0.022132	0.000490
**Error rate**						
CNN-QSAVNS	0.554545	0.772727	0.645833	0.628030	0.092692	0.008592
CNN-VNS	0.611364	0.756818	0.689583	0.695076	0.053358	0.002847
CNN-GA	0.770455	0.772727	0.772159	0.772727	0.000984	9.68E-07
CNN-PSO	0.569697	0.674242	0.626326	0.630682	0.045389	0.002060
CNN-ABC	0.636364	0.597727	0.616856	0.616667	0.014167	0.000201
CNN-BA	0.600000	0.706061	0.659280	0.665530	0.039570	0.001566
CNN-SCHO	0.568939	0.589394	0.586742	0.585606	0.013705	0.000188
CNN-EHO	0.752273	0.772727	0.767424	0.772348	0.008753	7.66E-05

Table 2 also reports the results of the indicator function expressed in terms of the error rate for CNN models optimized by the same set of metaheuristic techniques. QSAVNS achieved the lowest absolute error rate of 0.554545, while SCHO recorded the best mean error rate (0.586742), median error rate (0.585606), and the most favorable worst-case result (0.589394). Although GA did not achieve the top-performing absolute score, it demonstrated notable stability across repeated executions, indicating high robustness despite slightly lower overall optimization effectiveness.

Table 3 provides a comprehensive overview of the evaluation metrics corresponding to the CNN classifiers that achieved the best performance under different metaheuristic optimization methods. The findings show that the proposed QSAVNS algorithm generated a robust CNN model, reaching an overall classification accuracy of 0.445455, accompanied by consistently solid precision, recall (sensitivity), and F1-scores in most categories. Nevertheless, a clear pattern emerged in all the models, each showing a limited ability to accurately differentiate among the four stages of AD. This limitation underscores the need for additional methodological refinements, which are explored in the following sections of this study.

**Table 3:** Comprehensive assessment of the best-performing CNN models yielded by the optimization process.

**Approach**	**Metric**	**0**	**1**	**2**	**3**	**Accuracy**	**Macro ave**	**Weight ave**
CNN-QSAVNS	Precision	0.424501	0.4	0.5	0.470480	0.445455	0.448745	0.445873
	Recall	0.776042	0.035714	0.076667	0.85	0.445455	0.434606	0.445455
	F1-score	0.548803	0.065574	0.132948	0.605701	0.445455	0.338256	0.344218
CNN-VNS	Precision	0.490196	0	0	0.324691	0.388636	0.203722	0.216396
	Recall	0.651042	0	0	0.876667	0.388636	0.381927	0.388636
	F1-score	0.559284	0	0	0.473874	0.388636	0.258289	0.270399
CNN-GA	Precision	0	0.5	0	0.228311	0.229545	0.182078	0.179161
	Recall	0	0.008929	0	1	0.229545	0.252232	0.229545
	F1-score	0	0.017544	0	0.371747	0.229545	0.097323	0.088954
CNN-PSO	precision	0.425041	0.317073	0.446043	0.440901	0.430303	0.407265	0.405936
	recall	0.671875	0.038690	0.206667	0.783333	0.430303	0.425141	0.430303
	f1-score	0.520686	0.068966	0.282460	0.564226	0.430303	0.359084	0.361456
CNN-ABC	Precision	0.762887	0.457831	0	0.281879	0.363636	0.375649	0.402533
	Recall	0.385417	0.113095	0	0.98	0.363636	0.369628	0.363636
	F1-score	0.512111	0.181384	0	0.437826	0.363636	0.282830	0.294654
CNN-BA	Precision	0.498054	0	0	0.337469	0.4	0.208881	0.221586
	Recall	0.666667	0	0	0.906667	0.4	0.393333	0.4
	F1-score	0.570156	0	0	0.491863	0.4	0.265505	0.277650
CNN-SCHO	Precision	0.528708	0.406780	0.381720	0.385084	0.431061	0.425573	0.431624
	Recall	0.575521	0.071429	0.236667	0.843333	0.431061	0.431737	0.431061
	F1-score	0.551122	0.121519	0.292181	0.528736	0.431061	0.373389	0.377831
CNN-EHO	Precision	0.326613	0.327869	0.274510	0.047619	0.278788	0.244153	0.251683
	Recall	0.632813	0.178571	0.186667	0.03	0.278788	0.257013	0.278788
	F1-score	0.430851	0.231214	0.222222	0.036810	0.278788	0.230274	0.243064
	Samples	384	336	300	300			

**Figure 1:**
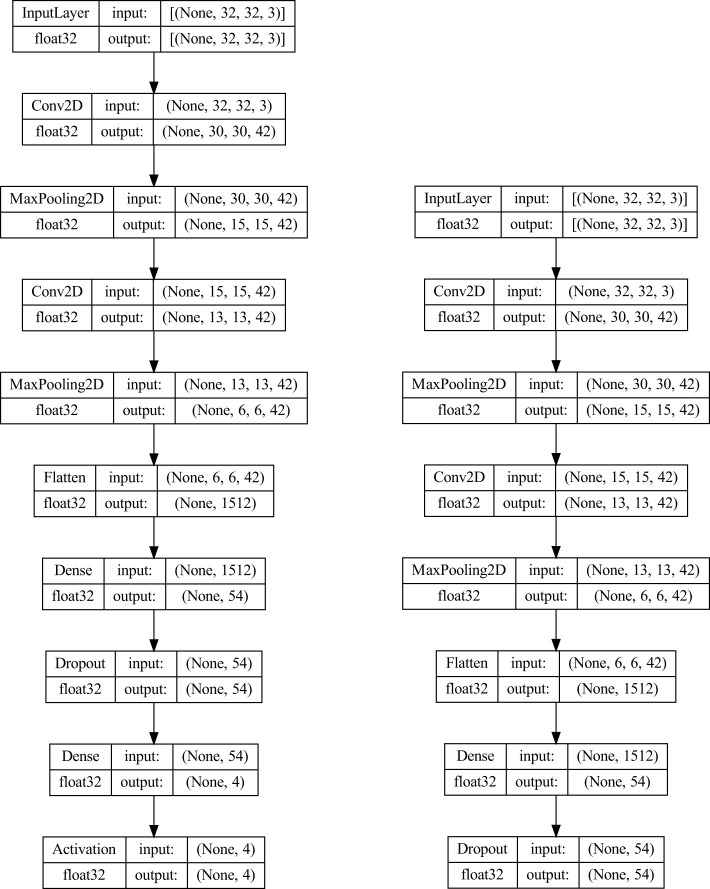
Optimized CNN architecture **(left)** and its truncated version **(right)**, where the final classification layers are removed to extract intermediate feature embeddings for L2 ensemble classification.

Figure 1 shows the architecture of the L1 CNN model with the best performance, along with its truncated counterpart. The left diagram shows the full CNN architecture used for end-to-end training and L1 optimization. The right diagram illustrates the truncated CNN obtained by removing the final classification layers. This model outputs intermediate feature embeddings that are subsequently used as input for the L2 ensemble classifiers.

### 5.2 L2 XGBoost

Table 4 presents a comparative evaluation of the XGBoost second-layer classifiers optimized using different metaheuristic algorithms, with the MCC serving as the primary evaluation metric. The proposed QSAVNS optimizer achieved the highest best-case result, achieving a peak MCC of 0.812047, while also demonstrating outstanding stability across other evaluation measures by recording the best mean values (0.796531) and median values (0.797621). These results highlight the robustness and reliability of QSAVNS as an optimization approach. Furthermore, QSAVNS achieved the strongest worst-case result (0.769870), while GA exhibited the lowest variability across repeated executions, indicating strong consistency in its optimization performance.

**Table 4:** Results of the objective and indicator function evaluations for L2 XGBoost optimization.

**Method**	**Best**	**Worst**	**Mean**	**Median**	**Std**	**Var**
CNN-XGB-QSAVNS	0.812047	0.769870	0.796531	0.797621	0.010066	0.000101
CNN-XGB-VNS	0.800002	0.718421	0.769789	0.775052	0.021384	0.000457
CNN-XGB-GA	0.800601	0.764087	0.784403	0.783531	0.009334	8.71E-05
CNN-XGB-PSO	0.799625	0.734070	0.779291	0.783115	0.015243	0.000232
CNN-XGB-ABC	0.771431	0.650755	0.720854	0.729989	0.034184	0.001169
CNN-XGB-BA	0.804570	0.752267	0.779033	0.780635	0.014211	0.000202
CNN-XGB-SCHO	0.799465	0.743217	0.779011	0.782598	0.013448	0.000181
CNN-XGB-EHO	0.800585	0.744689	0.780002	0.786002	0.018699	0.000350
**Error rate**						
CNN-XGB-QSAVNS	0.140909	0.172727	0.152475	0.151515	0.007588	5.76E-05
CNN-XGB-VNS	0.15	0.210606	0.172424	0.168182	0.015967	0.000255
CNN-XGB-GA	0.149242	0.176515	0.161515	0.162121	0.006966	4.85E-05
CNN-XGB-PSO	0.15	0.199242	0.165354	0.162121	0.011394	0.000130
CNN-XGB-ABC	0.171212	0.262121	0.209091	0.202273	0.025598	0.000655
CNN-XGB-BA	0.146212	0.185606	0.165505	0.164394	0.010642	0.000113
CNN-XGB-SCHO	0.15	0.192424	0.165455	0.162879	0.010110	0.000102
CNN-XGB-EHO	0.149242	0.191667	0.164949	0.160606	0.014142	0.000200

Table 4 also includes a comparative analysis based on the error rate indicator for the same XGBoost classifiers optimized with different metaheuristic methods. The most favorable result, corresponding to the lowest best-case error rate of 0.140909, was achieved by the proposed QSAVNS. In addition, QSAVNS outperformed competing algorithms by achieving the best mean and median error rates, measured at 0.152475 and 0.151515, respectively, confirming its high stability between independent runs. It also obtained the lowest worst-case error rate (0.172727) and demonstrated excellent consistency (second only to GA) in this evaluation scenario.

Table 5 provides detailed evaluation metrics for the top-performing L2 XGBoost classifiers optimized with different metaheuristic algorithms. Among them, the proposed QSAVNS achieved the best overall performance, yielding the most accurate CNN-XGBoost-based model with a maximum classification accuracy of 0.859091, while maintaining consistently high precision, recall (sensitivity), and F1-scores across all classes. An important observation from these results is that integrating XGBoost into the second layer of the framework significantly enhanced overall precision compared to standalone CNN models, notably improving the differentiation between AD stages. Nevertheless, as shown in the next subsection, the XGBoost classifiers were significantly outperformed by the LightGBM models implemented in the same layer.

**Table 5:** Comprehensive assessment of the best-performing L2 XGBoost models produced through the optimization process.

**Approach**	**Metric**	**0**	**1**	**2**	**3**	**Accuracy**	**Macro ave**	**Weight ave**
CNN-XGB-QSAVNS	Precision	0.844059	0.852349	0.824503	0.917722	0.859091	0.859658	0.858466
	Recall	0.888021	0.755952	0.83	0.966667	0.859091	0.860160	0.859091
	F1-score	0.865482	0.801262	0.827243	0.941558	0.859091	0.858886	0.857734
CNN-XGB-VNS	Precision	0.823671	0.828859	0.846690	0.906542	0.85	0.851441	0.849058
	Recall	0.888021	0.735119	0.81	0.97	0.85	0.850785	0.85
	F1-score	0.854637	0.779180	0.827939	0.937198	0.85	0.849738	0.848126
CNN-XGB-GA	Precision	0.854545	0.807927	0.844523	0.895062	0.850758	0.850514	0.849609
	Recall	0.856771	0.788690	0.796667	0.966667	0.850758	0.852199	0.850758
	F1-score	0.855657	0.798193	0.819897	0.929487	0.850758	0.850808	0.849682
CNN-XGB-PSO	Precision	0.837093	0.815873	0.838028	0.909938	0.85	0.850233	0.848460
	Recall	0.869792	0.764881	0.793333	0.976667	0.85	0.851168	0.85
	F1-score	0.853129	0.789555	0.815068	0.942122	0.85	0.849969	0.848522
CNN-XGB-ABC	Precision	0.810345	0.813725	0.819788	0.873846	0.828788	0.829426	0.827784
	Recall	0.856771	0.741071	0.773333	0.946667	0.828788	0.829461	0.828788
	F1-score	0.832911	0.775701	0.795883	0.9088	0.828788	0.828324	0.827181
CNN-XGB-BA	Precision	0.844221	0.799392	0.861818	0.915094	0.853788	0.855131	0.852917
	Recall	0.875	0.782738	0.79	0.97	0.853788	0.854435	0.853788
	F1-score	0.859335	0.790977	0.824348	0.941748	0.853788	0.854102	0.852713
CNN-XGB-SCHO	Precision	0.843038	0.80625	0.840278	0.911672	0.85	0.850309	0.848645
	Recall	0.867188	0.767857	0.806667	0.963333	0.85	0.851261	0.85
	F1-score	0.854942	0.786585	0.823129	0.936791	0.85	0.850362	0.848914
CNN-XGB-EHO	Precision	0.85533	0.809524	0.838488	0.896875	0.850758	0.850054	0.849285
	Recall	0.877604	0.758929	0.813333	0.956667	0.850758	0.851633	0.850758
	F1-score	0.866324	0.78341	0.825719	0.925806	0.850758	0.850315	0.849509
	Samples	384	336	300	300			

### 5.3 L2 LightGBM

Table 6 presents a comparative evaluation of the L2 LightGBM classification models optimized using several metaheuristic algorithms, with the MCC serving as the main objective function. Among the methods examined, the proposed QSAVNS once again proved to be the most effective optimizer, achieving the highest best-case MCC value of 0.860430 (tied with PSO) and achieving competitive results across other statistical indicators. The GA algorithm recorded the best worst-case performance (0.804565) and the highest mean (0.840211) and median (0.845163) MCC values. In addition, GA demonstrated exceptional consistency between independent runs, indicating minimal stochastic variability and high overall reliability.

**Table 6:** Results of the objective and indicator function evaluations for L2 LightGBM optimization.

**Method**	**Best**	**Worst**	**Mean**	**Median**	**Std**	**Var**
CNN-LGBM-QSAVNS	0.860430	0.735341	0.811917	0.815721	0.043857	0.001923
CNN-LGBM-VNS	0.840972	0.747018	0.797422	0.803751	0.028748	0.000826
CNN-LGBM-GA	0.859326	0.804565	0.840211	0.845163	0.015145	0.000229
CNN-LGBM-PSO	0.860430	0.761823	0.802577	0.804399	0.025077	0.000629
CNN-LGBM-ABC	0.827912	0.685182	0.733294	0.717491	0.043312	0.001876
CNN-LGBM-BA	0.841007	0.742303	0.789529	0.784178	0.031955	0.001021
CNN-LGBM-SCHO	0.845986	0.748065	0.792571	0.798346	0.027384	0.000750
CNN-LGBM-EHO	0.847054	0.712497	0.800066	0.814890	0.041080	0.001688
**Error rate**						
CNN-LGBM-QSAVNS	0.104545	0.198485	0.140808	0.137879	0.032887	0.001082
CNN-LGBM-VNS	0.118939	0.189394	0.151616	0.146970	0.021522	0.000463
CNN-LGBM-GA	0.105303	0.146212	0.119545	0.115909	0.011332	0.000128
CNN-LGBM-PSO	0.104545	0.178030	0.147727	0.146212	0.018717	0.000350
CNN-LGBM-ABC	0.128788	0.235606	0.199646	0.211364	0.032457	0.001053
CNN-LGBM-BA	0.118939	0.193182	0.157475	0.161364	0.023966	0.000574
CNN-LGBM-SCHO	0.115152	0.188636	0.155202	0.150758	0.020575	0.000423
CNN-LGBM-EHO	0.114394	0.215152	0.149646	0.138636	0.030779	0.000947

Table 6 also reports a comparative analysis of L2 LightGBM classifiers optimized using different metaheuristic strategies, this time based on the indicator function represented by the error rate. Among all algorithms tested, the proposed QSAVNS achieved the best overall outcome, with the lowest absolute error rate of 0.104545. While QSAVNS also produced competitive results for the remaining metrics, GA achieved the strongest worst-case performance (0.146212), along with the best mean error rates (0.119545) and median error (0.115909). Although GA did not reach the lowest absolute error, it exhibited the greatest consistency across repeated runs, demonstrating outstanding stability despite slightly weaker optimization performance compared to QSAVNS.

Table 7 provides detailed evaluation metrics for the top-performing L2 LightGBM classifiers optimized using the examined metaheuristic algorithms. The findings show that the QSAVNS-optimized model produced the most effective CNN-LightGBM configuration, achieving the highest overall classification accuracy of 0.895455 (matched by PSO) and consistently maintaining high precision, recall, and F1-scores in all evaluated classes. An important conclusion drawn from these results is that the integration of LightGBM into the second layer of the framework substantially enhanced overall accuracy compared to the standalone CNN architectures, while also improving the classification of individual stages of AD. Moreover, the LightGBM-based models in the second layer clearly outperformed their XGBoost counterparts, confirming the superior performance and adaptability of LightGBM within this hierarchical framework.

**Table 7:** Comprehensive assessment of the best-performing L2 LightGBM models obtained through the optimization process.

**Approach**	**Metric**	**0**	**1**	**2**	**3**	**Accuracy**	**Macro ave**	**Weight ave**
CNN-LGBM-QSAVNS	Precision	0.878173	0.880645	0.880795	0.94586	0.895455	0.896368	0.894781
	Recall	0.901042	0.8125	0.886667	0.99	0.895455	0.897552	0.895455
	F1-score	0.88946	0.845201	0.883721	0.967427	0.895455	0.896452	0.89461
CNN-LGBM-VNS	Precision	0.861111	0.849057	0.868687	0.951456	0.881061	0.882578	0.880297
	Recall	0.888021	0.803571	0.86	0.98	0.881061	0.882898	0.881061
	F1-score	0.874359	0.825688	0.864322	0.965517	0.881061	0.882471	0.880407
CNN-LGBM-GA	Precision	0.890374	0.854103	0.884106	0.952381	0.894697	0.895241	0.89381
	Recall	0.867188	0.83631	0.89	1	0.894697	0.898374	0.894697
	F1-score	0.878628	0.845113	0.887043	0.97561	0.894697	0.896598	0.894051
CNN-LGBM-PSO	Precision	0.878173	0.880645	0.880795	0.94586	0.895455	0.896368	0.894781
	Recall	0.901042	0.8125	0.886667	0.99	0.895455	0.897552	0.895455
	F1-score	0.88946	0.845201	0.883721	0.967427	0.895455	0.896452	0.89461
CNN-LGBM-ABC	Precision	0.866324	0.828571	0.850993	0.93949	0.871212	0.871345	0.869859
	Recall	0.877604	0.776786	0.856667	0.983333	0.871212	0.873597	0.871212
	F1-score	0.871928	0.801843	0.853821	0.960912	0.871212	0.872126	0.870196
CNN-LGBM-BA	Precision	0.868895	0.824451	0.876667	0.958333	0.881061	0.882087	0.879675
	Recall	0.880208	0.782738	0.876667	0.996667	0.881061	0.88407	0.881061
	F1-score	0.874515	0.803053	0.876667	0.977124	0.881061	0.88284	0.880134
CNN-LGBM-SCHO	Precision	0.862694	0.83792	0.888889	0.958065	0.884848	0.886892	0.884017
	Recall	0.867188	0.815476	0.88	0.99	0.884848	0.888166	0.884848
	F1-score	0.864935	0.826546	0.884422	0.97377	0.884848	0.887418	0.884328
CNN-LGBM-EHO	Precision	0.859649	0.861635	0.874576	0.954545	0.885606	0.887602	0.885115
	Recall	0.893229	0.815476	0.86	0.98	0.885606	0.887176	0.885606
	F1-score	0.876117	0.83792	0.867227	0.967105	0.885606	0.887093	0.885053
	Samples	384	336	300	300			

### 5.4 Visual comparative analysis

**Figure 2:**
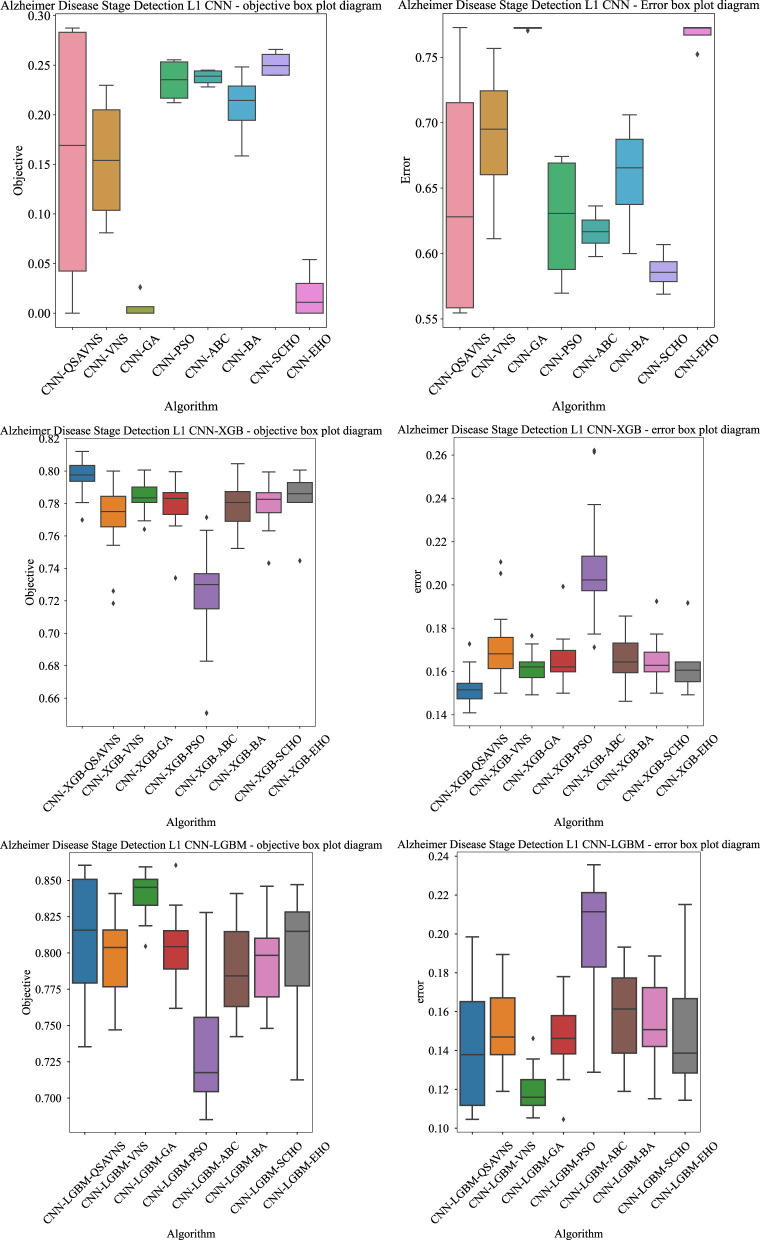
Scatter plots of the objective and indicator function results for the L1 CNN **(top)**, L2 XGBoost **(middle)**, and L2 LightGBM **(bottom)** experiments.

Figure 2 provides a detailed comparative analysis of different metaheuristic optimizers applied to fine-tune both hierarchical layers of the proposed classification framework for the identification of stages of AD. The evaluation covers three experimental setups: L1 CNN optimization (top row), L2 XGBoost optimization (middle row), and L2 LightGBM optimization (bottom row). To ensure statistical robustness, the performance of each optimizer was assessed in 30 independent runs, with the distributions of the MCC values illustrated through box plots. Thirty separate runs were necessary to obtain statistically meaningful results and reduce the influence of randomness inherent in metaheuristic algorithms (Talbi, 2009). These visualizations emphasize central tendencies (medians), variability, and asymmetry in distribution, offering a clear perspective on the balance between stability and exploratory dynamics exhibited by each optimization approach.

The results reveal that the proposed QSAVNS consistently achieved the highest best-case MCC values in all three experimental stages, confirming its strong global search capability. Additionally, the distribution of its results shows stable median values combined with slightly wider variance, indicating effective exploration that prevents premature convergence to local optima. This broader dispersion in MCC outcomes reflects a deliberate design trade-off, where superior best-run performance was obtained at the expense of slightly lower overall stability.

The box plots also summarize the statistical behavior of error rates collected from 30 independent optimization runs, illustrating both the central tendency and variability for each algorithm. These graphs are particularly valuable for assessing model generalization, lower median error rates coupled with narrower interquartile ranges correspond to more consistent and reliable predictive outcomes. Across all configurations, the proposed QSAVNS algorithm consistently achieved the lowest error rates, confirming its ability to preserve population diversity while efficiently exploiting promising regions of the search space. This balance effectively minimizes the risk of premature convergence and reduces misclassification tendencies.

**Figure 3:**
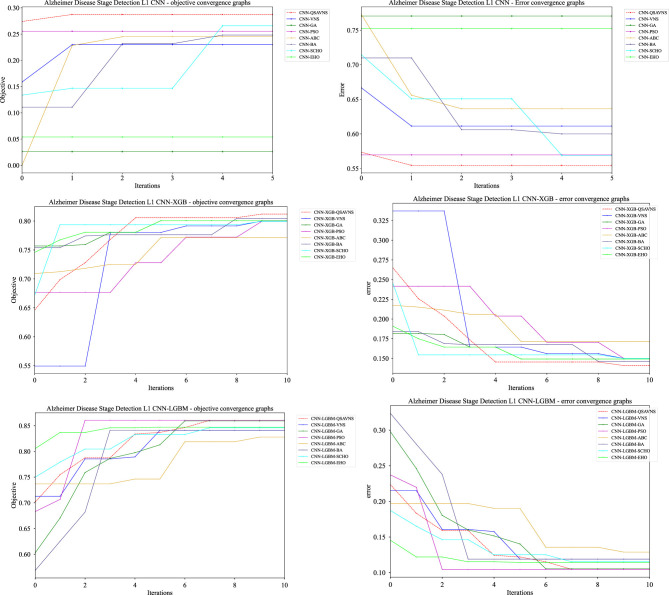
Convergence plots of the objective and indicator functions for the L1 CNN **(top)**, L2 XGBoost **(middle)**, and L2 LightGBM **(bottom)** experiments.

The complementary convergence plots shown in Figure 3 provide additional insight into the temporal progression of the optimization process, illustrating how each algorithm improves the objective function across successive iterations during their best-performing runs. The proposed QSAVNS demonstrates faster and more stable convergence behavior, which can be attributed to its adaptive features, including the QRL-based initialization and the integrated stagnation-aware rollback mechanism. These components foster a balanced interaction between exploration and exploitation, ensuring consistent progress throughout the search process. In contrast, alternative optimization algorithms often exhibit slower performance gains or early stagnation, reflecting reduced effectiveness in navigating complex, high-dimensional hyperparameter spaces.

In general, these findings highlight the decisive impact of the algorithmic structure on the efficiency of optimization in classification tasks. Approaches that maintain population diversity, enable structured exploration of neighboring regions, and dynamically regulate diversity during the search exert a significant influence on the convergence rate, the quality of the solution and the reproducibility. Such characteristics are particularly critical in real-world applications, where sensitivity to initialization settings and variations in input data can substantially affect predictive stability and reliability.

**Figure 4:**
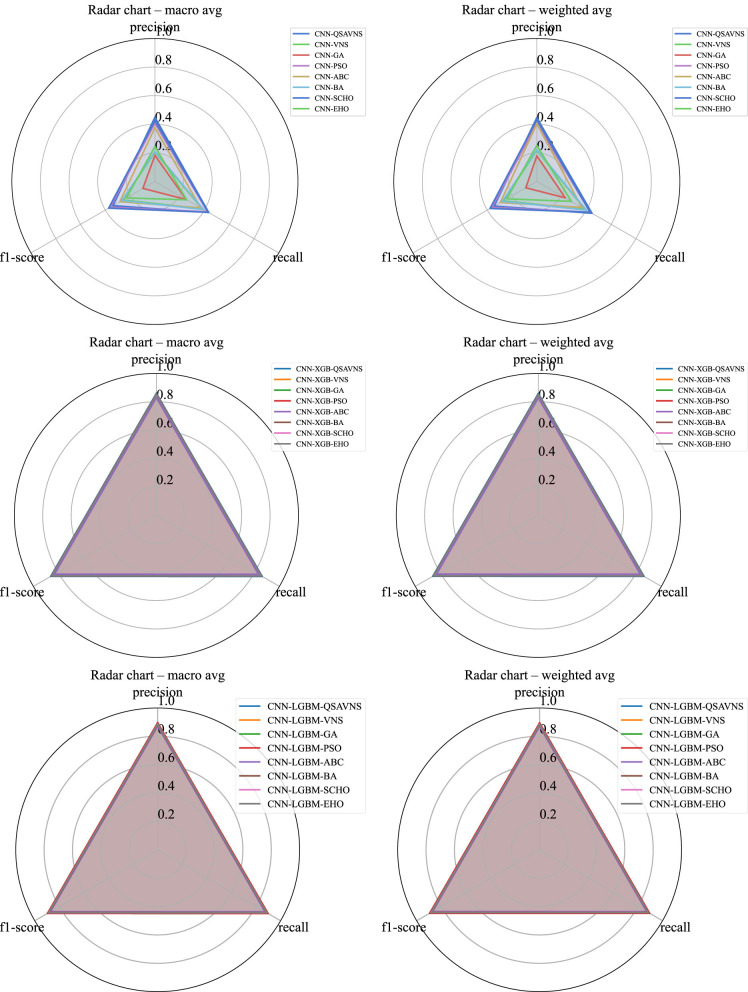
Radar charts illustrating macro and weighted-average evaluation metrics for the L1 CNN **(top)**, L2 XGBoost **(middle)**, and L2 LightGBM **(bottom)** simulations.

Figure 4 presents radar charts that summarize both macro and weighted-averaged results, offering a comprehensive depiction of classifier performance across multiple evaluation metrics. The macro-average treats all classes equally, making it particularly useful for assessing a model's ability to correctly recognize minority classes, an especially challenging aspect in scenarios characterized by class imbalance. In contrast, the weighted average accounts for the frequency of the classes, producing a metric that reflects the overall class distribution within the dataset and assigns greater importance to the performance of the majority classes.

Displaying these two perspectives side by side reveals the inherent trade-offs among the different optimization algorithms. Models that achieve high weighted-average values may still face challenges in generalizing to minority classes, whereas those demonstrating stronger macro-average results tend to exhibit greater resilience and robustness in imbalanced data contexts. Together, the radar plots provide a complementary means of analysis, supporting a more nuanced evaluation of generalization capability, fairness, and reliability of the classifiers optimized using the metaheuristic approaches examined.

### 5.5 Discussion

In the first stage of the proposed framework, CNN functions as a feature extraction engine, capturing hierarchical and discriminative representations from MRI data. However, the conventional practice of relying on a dense output layer for classification within CNN architectures often fails to fully exploit the richness of the extracted features, resulting in suboptimal predictive performance. Replacement of this terminal layer with advanced ensemble classifiers substantially enhances the accuracy and robustness of the model. Following this principle, the proposed framework substitutes the CNN's dense layer with XGBoost and LightGBM classifiers, both of which demonstrate superior performance relative to the baseline of the dense layer. By integrating CNN-based deep feature learning with gradient enhancement techniques for classification and refining both levels through metaheuristic-driven hyperparameter optimization, the framework effectively combines the strengths of deep representation learning and ensemble-based decision making. This hybrid configuration leads to marked improvements in predictive accuracy and computational efficiency for the classification of stages of Alzheimer's disease, and both L2 models outperform the CNN of the baseline in terms of classification accuracy.

Analysis of the fitness function, expressed through the MCC, shows that models incorporating LightGBM in the second layer (L2) consistently outperform those utilizing XGBoost. Both ensemble-based configurations exceed the CNN baseline in the first layer (L1). The box plot analyzes further reveal that L2 LightGBM achieves the highest median and maximum MCC values, confirming its superior capacity to identify subtle and complex discriminative features critical for accurate stage differentiation. Additionally, the convergence patterns of LightGBM display smooth and stable optimization behavior across all metaheuristic algorithms. This effect is most evident when optimized using QSAVNS, where LightGBM attains the highest recorded MCC values, substantially reinforcing the discriminative capacity of the framework for this clinically important classification problem.

A similar trend is observed for the indicator function, represented by the error rate, where lower values correspond to better performance. Across all three experimental configurations, LightGBM in the L2 layer consistently achieves the lowest error rates, often by a significant margin. The best-performing LightGBM configuration, CNN-LGBM-QSAVNS, achieved the highest overall classification accuracy of 0.895455. Although XGBoost in L2 also produced strong and competitive results, LightGBM demonstrated superior stability and generalization among different metaheuristic optimizers. Taken together, these results establish LightGBM as the most suitable choice for the second layer of the proposed AD stage classification framework, combining high predictive accuracy, low error rates, and consistent performance, qualities essential for reliable clinical implementation.

From the perspective of general optimization theory, the coupling of metaheuristic optimization with ensemble learning aligns with the principles of adaptive search in complex, high-dimensional search spaces. Metaheuristic algorithms are particularly effective in navigating non-convex and discontinuous objective spaces, where gradient-based or deterministic tuning methods often fail. When metaheuristics are combined with ensemble models like XGBoost and LightGBM in this study (which themselves rely on aggregating multiple weak learners), the optimization process benefits from complementary mechanisms of exploration and exploitation at both the parameter-search and decision-fusion levels. This synergy is consistent with the NFL theorem, which suggests that performance gains arise not from universally optimal algorithms, but from well-matched combinations of optimization strategies and learning models tailored to a specific problem domain.

To facilitate reproducibility of experimental results, the hyperparameter configurations for the best-performing models, L1 CNN, L2 XGBoost, and L2 LightGBM, are summarized in Table 8.

Table 8Selected hyperparameter configurations for the best-performing L1 CNN, L2 XGBoost, and L2 LightGBM architectures.

**Table:** 

**L1 CNN**	**LR**	**Drop**	**Epochs**	**CNN-L**	**Dense-L**	**CNN1**	**CNN2**	**DL1**	**DL2**
CNN-QSAVNS	0.0013	0.1618	29	1	1	43	N/A	96	N/A
CNN-VNS	0.0002	0.1913	30	1	1	32	N/A	41	N/A
CNN-GA	0.001	0.0761	20	1	1	68	N/A	51	N/A
CNN-PSO	0.0009	0.0857	30	1	1	48	N/A	96	N/A
CNN-ABC	0.0005	0.1941	26	1	1	54	N/A	89	N/A
CNN-BA	0.0005	0.1407	30	2	1	69	63	90	N/A
CNN-SCHO	0.0021	0.05	30	1	1	39	N/A	36	N/A
CNN-EHO	0.0009	0.1372	13	2	2	52	69	93	32

**Table:** 

**L2 XGBoost**	**LR**	**MCW**	**Sub-sample**	**Co-sample**	**Max depth**	γ
CNN-XGB-QSAVNS	0.9	1	1	0.8418	5	0.4992
CNN-XGB-VNS	0.9	1.4154	0.9191	1	5	0.1318
CNN-XGB-GA	0.9	1	0.9107	1	5	0.6213
CNN-XGB-PSO	0.8547	1	0.8005	1	5	0.1726
CNN-XGB-ABC	0.9	2.3408	0.7818	1	5	0.3861
CNN-XGB-BA	0.9	1	1	0.9072	5	0.0476
CNN-XGB-SCHO	0.9	1.2104	0.883	0.6711	5	0
CNN-XGB-EHO	0.9	1	1	1	5	0.1096

**Table:** 

**L2 LightGBM**	**Rounds**	**Max depth**	**Leaves**	**MCW**	**FF**	**BF**	**MSG**	λ **L1**	λ **L2**	**lr**
CNN-LGBM-QSAVNS	20	10	10	4	0.9	1	0.1	0	0	0.9
CNN-LGBM-VNS	20	9	10	3	0.9	0.5	0.0022	0	1.0995	0.898
CNN-LGBM-GA	20	8	10	2	0.9	0.614	0.0792	0	0.1935	0.8966
CNN-LGBM-PSO	20	10	10	4	0.9	0.5	0.0997	0	0	0.9
CNN-LGBM-ABC	20	10	10	1	0.9	0.7564	0.0678	0	0.7786	0.8659
CNN-LGBM-BA	20	6	10	1	0.9	0.5	0.0831	0.3253	0.3123	0.9
CNN-LGBM-SCHO	20	10	10	2	0.8696	0.8579	0.0796	0	0	0.8734
CNN-LGBM-EHO	20	10	10	1	0.9	1	0.1	1.2477	0	0.9

## 6 Validation and interpretation

### 6.1 Comparisons to baselines

To further evaluate the performance of the proposed framework, the best second-layer (L2) models were compared with a set of well-established benchmark classifiers. The benchmark suite included a multi-layer perceptron (MLP), decision tree (DT) (de Ville, 2013), k-nearest neighbors (KNN) (Kramer, 2013), random forest (RF) (Breiman, 2001), and several boosting algorithms, AdaBoost, CatBoost, plain LightGBM (Ke et al., 2017), and plain XGBoost (Chen and Guestrin, 2016), as well as a deep CNN (Gu et al., 2018). All baseline classifiers were trained and tested using their default hyperparameter configurations, and the resulting evaluation metrics are summarized in Table 9.

**Table 9:** Comparison of the best-performing L2 models with baseline classification algorithms.

**Approach**	**Metric**	**0**	**1**	**2**	**3**	**Accuracy**	**Macro ave**	**Weight ave**
CNN-XGB-QSAVNS	Precision	0.844059	0.852349	0.824503	0.917722	0.859091	0.859658	0.858466
	Recall	0.888021	0.755952	0.83	0.966667	0.859091	0.860160	0.859091
	F1-score	0.865482	0.801262	0.827243	0.941558	0.859091	0.858886	0.857734
CNN-LGBM-QSAVNS	Precision	0.878173	0.880645	0.880795	0.94586	0.895455	0.896368	0.894781
	Recall	0.901042	0.8125	0.886667	0.99	0.895455	0.897552	0.895455
	F1-score	0.88946	0.845201	0.883721	0.967427	0.895455	0.896452	0.89461
MLP	Precision	0.633609	0.395189	0.408537	0.704142	0.543182	0.535369	0.537798
	Recall	0.598958	0.342262	0.446667	0.793333	0.543182	0.545305	0.543182
	F1-score	0.615797	0.366826	0.426752	0.746082	0.543182	0.538864	0.539068
DT	Precision	0.505	0.329446	0.394265	0.647651	0.468182	0.469091	0.467567
	Recall	0.526042	0.33631	0.366667	0.643333	0.468182	0.468088	0.468182
	F1-score	0.515306	0.332842	0.379965	0.645485	0.468182	0.4684	0.467688
KNN	Precision	0.527542	0.353801	0.407563	0.738806	0.503788	0.506928	0.504064
	Recall	0.648438	0.360119	0.323333	0.66	0.503788	0.497972	0.503788
	F1-score	0.581776	0.356932	0.360595	0.697183	0.503788	0.499121	0.500503
RF	Precision	0.594937	0.423567	0.460993	0.68693	0.548485	0.541607	0.541781
	Recall	0.611979	0.395833	0.433333	0.753333	0.548485	0.54862	0.548485
	F1-score	0.603338	0.409231	0.446735	0.718601	0.548485	0.544476	0.544533
LGBM	Precision	0.638356	0.387755	0.433657	0.739274	0.548485	0.549761	0.55098
	Recall	0.606771	0.395833	0.446667	0.746667	0.548485	0.548984	0.548485
	F1-score	0.622163	0.391753	0.440066	0.742952	0.548485	0.549233	0.549579
CB	Precision	0.606299	0.389571	0.451724	0.708978	0.543939	0.539143	0.539337
	Recall	0.601563	0.377976	0.436667	0.763333	0.543939	0.544885	0.543939
	F1-score	0.603922	0.383686	0.444068	0.735152	0.543939	0.541707	0.541356
XGBoost	Precision	0.594737	0.400612	0.439344	0.737013	0.543939	0.542926	0.542342
	Recall	0.588542	0.389881	0.446667	0.756667	0.543939	0.545439	0.543939
	F1-score	0.591623	0.395173	0.442975	0.746711	0.543939	0.544121	0.543081
AdaBoost	Precision	0.532632	0.396154	0.408889	0.641667	0.514394	0.494835	0.494549
	Recall	0.658854	0.306548	0.306667	0.77	0.514394	0.510517	0.514394
	F1-score	0.589057	0.345638	0.350476	0.7	0.514394	0.496293	0.498087
CNN	Precision	0.489583	0.388646	0.445545	0.709265	0.517424	0.50826	0.503809
	Recall	0.734375	0.264881	0.3	0.74	0.517424	0.509814	0.517424
	F1-score	0.5875	0.315044	0.358566	0.724307	0.517424	0.496354	0.49721
	Samples	384	336	300	300			

Although the benchmark models demonstrated generally solid accuracy, the proposed L2 architectures consistently outperformed them in all evaluation criteria, achieving superior class-wise results and substantially higher overall accuracy. The best performing model, CNN-LGBM-QSAVNS, achieved an accuracy of 0.895455, followed by CNN-XGB-QSAVNS with 0.859091. In comparison, the best-performing baseline models, plain LightGBM and random forest, reached considerably lower accuracies of 0.548485 under the same experimental conditions.

### 6.2 Statistical analysis

Although comparative analysis of optimization algorithms can offer valuable information, conclusions drawn from a single execution are inherently unreliable. The stochastic nature of metaheuristic methods introduces significant variability between runs, rendering single-instance results insufficient to accurately evaluate overall performance. To mitigate randomness and improve the robustness of the evaluation, each algorithm in this study was executed 30 times with independent random seeds. This procedure yielded comprehensive distributions of the results, providing a statistically sound foundation for comparison. Such a multi-run evaluation protocol not only strengthens statistical validity but also enables more accurate identification of performance trends. In addition, this methodology aligns with widely accepted best practices for benchmarking metaheuristic algorithms (LaTorre et al., 2021), thus improving both the credibility and reproducibility of the study's results.

Statistical procedures for determining the significance of performance differences among groups are generally divided into parametric and non-parametric tests. The choice between them depends on assumptions such as the independence of observations, normality of the data distribution, and equality of variances between groups (homoscedasticity) (LaTorre et al., 2021). Independence was ensured by initializing each algorithmic run with a distinct random seed, preventing inter-run dependencies. Homoscedasticity was examined using the Levene test (Schultz, 1985), which produced a *p*-value of 0.88 for all experimental results, indicating that there are no statistically significant variance differences between the groups. The assumption of normality was then tested with the Shapiro–Wilk test (Shapiro and Francia, 1972). Since all computed *p*-values were below the standard 0.05 threshold, the null hypothesis of normality was rejected, confirming that the data did not satisfy the conditions required for parametric statistical tests.

Given the violation of normality, subsequent analyzes employed non-parametric methods. Specifically, the Wilcoxon signed-rank test (Woolson, 2005) was applied to perform pairwise comparisons between the proposed QSAVNS and each of the competing optimization algorithms. The resulting *p*-values, listed in Table 10, were all below the conventional significance level of α = 0.05, confirming that QSAVNS achieved statistically significant improvements over all alternative approaches.

**Table 10:** Wilcoxon test results comparing the QSAVNS algorithm with alternative optimizers across the three experimental configurations.

**QSAVNS vs. others**	**VNS**	**GA**	**PSO**	**ABC**	**BA**	**SCHO**	**EHO**
L1 CNN	0.034	0.027	0.041	0.042	0.041	0.044	0.037
L2 XGBoost	0.029	0.039	0.035	0.023	0.033	0.034	0.036
L2 LightGBM	0.037	0.044	0.041	0.023	0.032	0.035	0.039

These results provide strong empirical evidence that the superior performance of QSAVNS is not due to random fluctuations or sampling bias. Instead, they confirm a consistent and meaningful advantage across all three experimental configurations, underscoring both the robustness and the practical effectiveness of the proposed enhanced optimization method.

### 6.3 Best model interpretation

The practical importance of machine learning classifiers extends beyond achieving high predictive accuracy to encompass the interpretability and transparency of their internal decision-making processes. Interpretability provides crucial insights into the mechanisms underlying algorithmic predictions, allowing the detection of hidden biases, the identification of key predictive features, and the refinement of analytical workflows. This transparency is especially valuable for improving data acquisition, feature engineering, and preprocessing procedures, ultimately contributing to the development of more reliable and trustworthy models. In image-based analysis, specifically, understanding which features exert the greatest influence on classification outcomes improves both the explanatory depth and the practical applicability of the model. However, as machine learning architectures, particularly DL systems, grow increasingly complex, achieving interpretability becomes substantially more difficult. The deeper and more intricate the model, the less transparent its internal reasoning tends to be, making it challenging to trace errors, identify sources of bias, or align algorithmic logic with human understanding. This opacity can erode trust in automated systems, particularly in high-stakes domains such as healthcare, where accountability and interpretability are essential.

To address these challenges, the present study employed SHAP (SHapley Additive exPlanations) (Lundberg and Lee, 2017) within the proposed two-tier classification framework. SHAP offers a unified and theoretically grounded approach to interpreting model predictions by quantifying the contribution of each input feature, thereby clarifying how specific factors influence decision outcomes. In this research, the standard SHAP methodology was applied directly to the output of models optimized with the QSAVNS algorithm, without any algorithmic modifications. This interpretive layer proved critical for identifying the most influential features that affect classification performance, an especially important consideration in clinical contexts, where understanding the rationale behind predictions is as vital as their accuracy. The kernel explainer variant of SHAP was used to examine the proposed multi-level system, effectively isolating the relative contributions of the CNN-based feature extraction stage, the ensemble classifiers, and the metaheuristic optimization process. This approach provided a comprehensive and transparent understanding of how the hybrid framework generates its final predictions.

**Figure 5:**
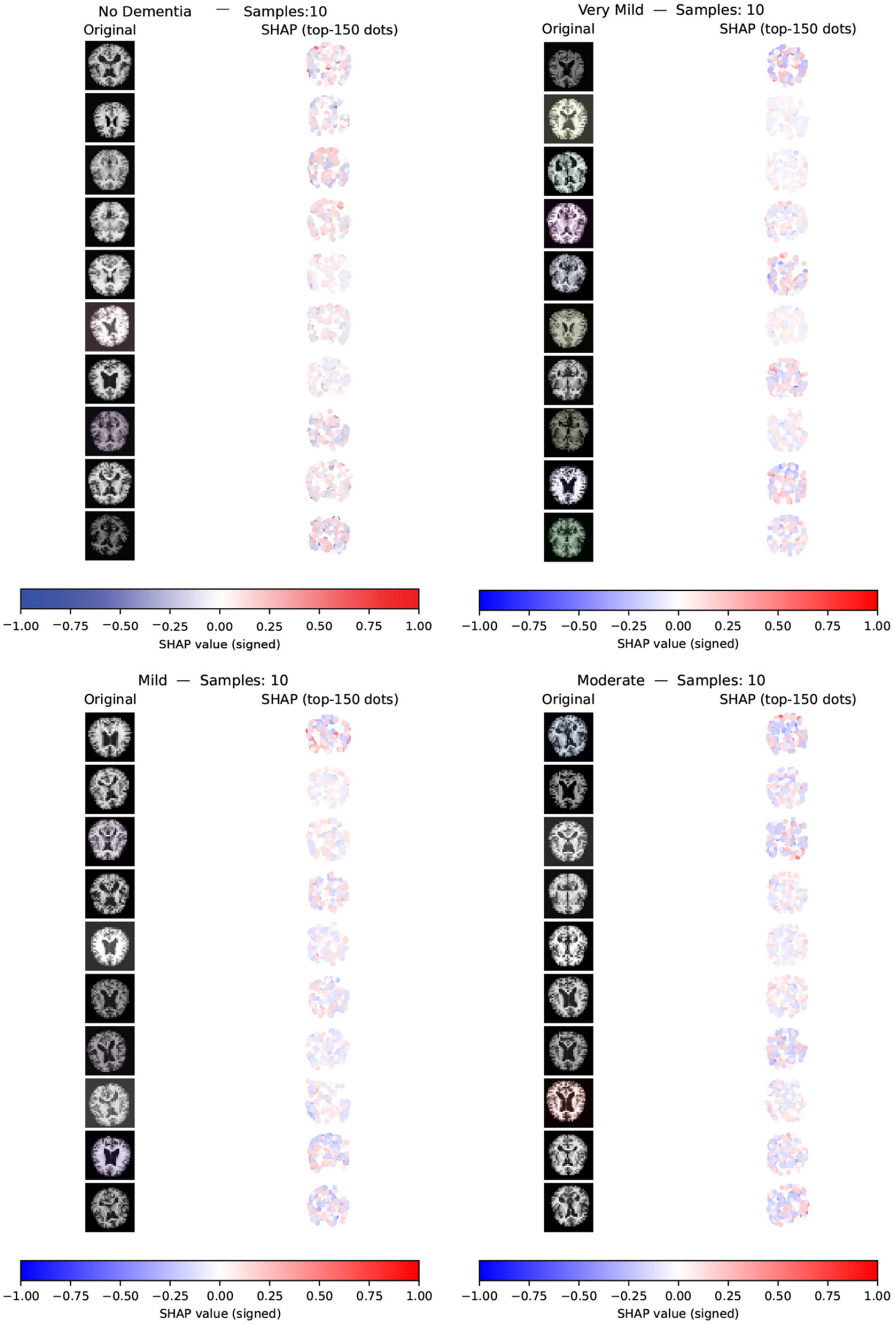
Best-performing QSAVNS-optimized multi-class LightGBM model, showing results for Class 0 (No Dementia), Class 1 (Very Mild), Class 2 (Mild), and Class 3 (Moderate).

For the interpretation of CNN-based models, the deep explainer variant of SHAP was used to identify and visualize the most influential features in the convolutional layers, offering a detailed representation of the internal reasoning of the model. The results of the multi-class classification analysis are illustrated in Figure 5, which contrasts interpretations obtained from the deep SHAP explainer with those generated using the kernel-based SHAP method applied to the XGBoost and LightGBM multi-tier frameworks. These comparative visualizations clarify how individual input features contribute across different layers of the models, providing a more comprehensive and transparent understanding of their underlying predictive mechanisms.

SHAP visualizations for the four classes of AD reveal distinct contribution patterns that evolve with disease progression. For the No Dementia class, the SHAP value distribution is relatively balanced, encompassing both positive and negative contributions. This balance suggests that the model's decisions for this class rely on a wide and diverse range of features, some reinforcing and others opposing the non-dementia classification, indicating that the decision-making process is based on varied and diffuse characteristics. In contrast, the Very Mild and Mild Dementia classes display more compact SHAP clusters, signifying that a smaller set of features exerts a stronger influence on the predictions. This concentration is in line with clinical expectations, as the early stages of Alzheimer's are marked by subtle, localized structural or functional alterations that serve as emerging differentiation signals. For the Moderate Dementia class, SHAP distributions become markedly polarized, revealing that a limited number of dominant features almost exclusively drive the model's predictions as the disease advances.

Together, these results illustrate a progression-dependent landscape of significance. During the earlier stages of the disease, the predictive reasoning of the model is based on a broad and heterogeneous collection of features, reflecting the inherent diagnostic ambiguity associated with early detection of Alzheimer's. As the condition progresses, the focus of the model narrows to a smaller group of highly discriminative features, consistent with the emergence of more distinct and stable pathological patterns. From a clinical perspective, this evolution mirrors real-world diagnostic challenges, while early-stage Alzheimer's detection depends on the recognition of subtle and diffuse anomalies, advanced stages present more pronounced and easily identifiable biomarkers. Consequently, SHAP-based interpretive analysis not only validates the predictive reliability of the proposed framework but also provides valuable clinical insight into which neuroimaging characteristics have the greatest diagnostic relevance in different phases of the progression of Alzheimer's disease.

## 7 Conclusion

Integration of accurate stage-classification models into clinical workflows has significant policy and operational implications for the management of AD. Early and precise stratification of patients across disease stages enables clinicians and healthcare systems to make more informed decisions regarding treatment planning, intensity of care, and allocation of specialized, often scarce, resources. Predictive insights generated by ML models can support the prioritization of critical interventions such as advanced neuroimaging, neuropsychological assessments, or enrollment in clinical trials, particularly in settings with limited diagnostic capacity and funding. For policymakers, these technologies provide the foundation for adaptive care pathways that dynamically align healthcare delivery with patient-specific needs, thus improving both system efficiency and individual patient outcomes.

Beyond direct clinical applications, the deployment of such classification frameworks has broader long-term implications for the design and strategy of healthcare systems. Reliable prediction of AD stages can contribute to the creation of standardized evidence-based protocols for diagnosis, monitoring, and transitions between different levels of care. This reduces clinical variability, improves diagnostic consistency, and promotes equitable access to specialized treatments. In addition, longitudinal datasets produced by AI-enabled diagnostic systems can inform national dementia strategies, guide preventive interventions for at-risk populations, and support the development of reimbursement models that emphasize measurable health outcomes.

However, the realization of these benefits depends on the establishment of comprehensive policy frameworks that address the ethical, legal, and technical challenges associated with the integration of AI in healthcare. Key priorities include enforcing rigorous standards for data privacy and governance, ensuring algorithmic transparency and interpretability, and implementing robust training programs to prepare clinicians, data scientists, and administrators to critically assess and safely use AI-based tools. Only through such safeguards can the integration of intelligent stage-classification systems achieve both clinical reliability and public trust, ultimately fostering a responsible and sustainable application of AI in real-world medical environments.

Accurate classification methods are essential to understand and manage the progression of AD, as they allow for precise staging that directly informs therapeutic strategies, supports continuous monitoring and improves patient quality of life. Because clinical differentiation between early, intermediate, and advanced stages is vital for determining both the timing and intensity of interventions, reliable stratification tools play a key role in guiding treatment selection, prioritizing clinical resources, and informing long-term prognostic decisions. In addition, advanced classification frameworks have the capability to uncover subtle, multidimensional patterns within neuroimaging and clinical datasets that are often undetectable using conventional diagnostic approaches.

To address these challenges, this study proposed a two-tier hybrid framework that integrates CNNs for feature extraction with ensemble learning classifiers, specifically XGBoost and LightGBM, for AD stage prediction. The performance of the model was further enhanced through metaheuristic-driven hyperparameter optimization, utilizing a customized variant of the VNS algorithm specifically adapted for this purpose. The framework was evaluated on publicly available AD datasets in a multi-class classification setting aimed at distinguishing among distinct disease stages. The best-performing configuration, CNN-based feature extraction combined with LightGBM classification optimized through the proposed QSAVNS algorithm, achieved a maximum precision of 89.55%, representing a significant improvement in both predictive accuracy and stage identification reliability.

Comprehensive statistical analyzes validated the superiority of the proposed approach compared to standard VNS and other widely used metaheuristic optimization algorithms. To enhance interpretability and model transparency, a SHAP analysis was applied to the best-performing configuration. Feature vectors extracted from the CNN's post-dropout layer were entered into the LightGBM classifier and SHAP values were calculated to quantify the contribution of individual features to the model's predictions, thus elucidating its internal decision-making process.

The proposed methodology introduces several distinct advantages. The tailored QSAVNS optimizer consistently outperformed existing metaheuristic algorithms, while the dual-layer architecture achieved substantially higher classification accuracy than the baseline CNN models without introducing excessive computational complexity. From a clinical point of view, this hybrid model shows great potential for real-world deployment in the diagnosis and management of AD. Accurate stage classification facilitates earlier detection, more targeted treatment planning, and improved prognostic assessment, ultimately contributing to the development of more personalized, effective, and adaptive care strategies for individuals affected by Alzheimer's disease.

Nevertheless, several limitations of this study should be recognized. The comparative evaluation included a relatively narrow set of optimization algorithms and was limited by modest population sizes and iteration counts. Future investigations will seek to address these constraints by broadening the scope of metaheuristic techniques considered and performing larger-scale experimental analyses, depending on the availability of greater computational resources. Such expansions are expected to yield deeper insights and more broadly generalizable findings. Furthermore, the proposed QSARSA algorithm demonstrates strong potential for adaptation to a wide range of ML tasks that demand sophisticated hyperparameter optimization. Extending this framework to handle real-time or streaming neuroimaging data also represents a promising avenue to advance clinical decision-support systems that aim to improve the diagnosis and treatment of AD.

## Data Availability

Publicly available datasets were analyzed in this study. This data can be found here: https://www.kaggle.com/datasets/aryansinghal10/alzheimers-multiclass-dataset-equal-and-augmented.

## Footnotes

1
https://www.who.int/news-room/fact-sheets/detail/dementia

2
https://www.kaggle.com/datasets/aryansinghal10/alzheimers-multiclass-dataset-equal-and-augmented
